# Differentiation between Vergence and Saccadic Functional Activity within the Human Frontal Eye Fields and Midbrain Revealed through fMRI

**DOI:** 10.1371/journal.pone.0025866

**Published:** 2011-11-02

**Authors:** Yelda Alkan, Bharat B. Biswal, Tara L. Alvarez

**Affiliations:** 1 Department of Biomedical Engineering, New Jersey Institute of Technology, Newark, New Jersey, United States of America; 2 Department of Radiology, University of Medicine and Dentistry of New Jersey, Newark, New Jersey, United States of America; Kyushu University, Japan

## Abstract

**Purpose:**

Eye movement research has traditionally studied solely saccade and/or vergence eye movements by isolating these systems within a laboratory setting. While the neural correlates of saccadic eye movements are established, few studies have quantified the functional activity of vergence eye movements using fMRI. This study mapped the neural substrates of vergence eye movements and compared them to saccades to elucidate the spatial commonality and differentiation between these systems.

**Methodology:**

The stimulus was presented in a block design where the ‘off’ stimulus was a sustained fixation and the ‘on’ stimulus was random vergence or saccadic eye movements. Data were collected with a 3T scanner. A general linear model (GLM) was used in conjunction with cluster size to determine significantly active regions. A paired *t*-test of the GLM beta weight coefficients was computed between the saccade and vergence functional activities to test the hypothesis that vergence and saccadic stimulation would have spatial differentiation in addition to shared neural substrates.

**Results:**

Segregated functional activation was observed within the frontal eye fields where a portion of the functional activity from the vergence task was located anterior to the saccadic functional activity (z>2.3; p<0.03). An area within the midbrain was significantly correlated with the experimental design for the vergence but not the saccade data set. Similar functional activation was observed within the following regions of interest: the supplementary eye field, dorsolateral prefrontal cortex, ventral lateral prefrontal cortex, lateral intraparietal area, cuneus, precuneus, anterior and posterior cingulates, and cerebellar vermis. The functional activity from these regions was not different between the vergence and saccade data sets assessed by analyzing the beta weights of the paired *t*-test (p>0.2).

**Conclusion:**

Functional MRI can elucidate the differences between the vergence and saccade neural substrates within the frontal eye fields and midbrain.

## Introduction

During natural viewing conditions we use a combination of version (saccade and smooth pursuit) and vergence (convergence and divergence) eye movements [1]. Saccades and smooth pursuit are conjugate movements where the eyes move in tandem. Vergence is the inward (convergence) and outward (divergence) rotation of the eyes to view objects at different spatial depths. With the increased presence of smart phones and tablets, our society has become more dependent on small interface devices. In addition, the use of 3D stereoscopic displays for computers, which stimulates vergence, is becoming common for vocational and recreational activities. Hence, the combination of vergence and saccadic movements especially for near viewing applications are prevalent within our activities of daily living. Currently, clinicians are reporting an increase in visual symptoms associated with sustained near viewing tasks where vergence is utilized [2], [3], [4]. However, fewer studies have been conducted on vergence compared to saccadic eye movements [5]. Thus, a detailed functional MRI study quantifying the neural substrates of vergence movements in comparison with saccadic movements is warranted.

In the last five years alone, numerous reviews have summarized the state of the art in saccadic research describing: eye movement behavior in humans [6]; single cell electrophysiology research on primates [7]; case reports of humans with lesions [8] and functional imaging studies [9]. Many primate studies have investigated the cellular responses from saccadic stimuli [1], [7]. Yet, many of those studies did not include a dynamic vergence stimulus such as a step change in disparity. Hence, it is unclear whether the cells that encode for saccades are also tuned for disparity – the input stimulus for vergence.

There are a few investigations that have specifically sought to study the cortical location of the disparity vergence signal in primates where cells have been identified that modulate their activity for vergence but not for saccadic stimuli. Gamlin and Yoon (2000) report activity from cells that modulate their behavior with saccadic stimuli within the anterior bank of the arcuate sulcus which is defined as part of the frontal eye fields [10]. Gamlin and Yoon report distinct cells located anterior to the saccadic cells that modulate their activity with vergence stimuli but not with saccadic stimuli [11]. Within the subcortical regions, distinct cells that encode for vergence but not for saccadic / smooth pursuit movements have also been identified within the midbrain, specifically within the mesencephalic reticular formation and dorsal lateral to the oculomotor nucleus [12], [13], [14], [15], [16], [17].

Behavioral eye movement data support interactions between vergence and saccadic eye movements [18], [19], [20], [21], [22]. Studies report that peak vergence velocity is greater when it is accompanied by a saccadic movement [18], [23] and that saccadic peak velocity is slower when it is accompanied by a vergence movement [24]. Several studies support that the parietal lobe modulates its activity for saccadic [25], [26], [27] and disparity (the input to vergence) [28], [29] stimulation. The cerebellum has also been implicated in error processing for motor learning for both saccadic [30], [31], [32] and vergence [33], [34], [35] movements.

Based upon the aforementioned data from both cellular and behavioral studies of the saccadic and vergence systems, we hypothesize that the neural substrates involved in initiating vergence and saccade eye movements will have shared neural resources within the parietal and cerebellar regions. We further hypothesize that differentiation will be observed within the frontal eye fields and midbrain regions. The aim of this study is to compare the vergence and saccade neural resources using the blood oxygenation level dependent signal from fMRI to systematically study the spatial differences and commonality between the neural substrates used to elicit saccade and vergence eye movements.

## Methods

### Subjects

The New Jersey Institute of Technology (NJIT) and University of Medicine and Dentistry of New Jersey (UMDNJ) Institution Review Board (IRB) approved this study. All subjects signed written informed consent forms approved by the NJIT and UMDNJ IRB in accordance with the Declaration of Helsinki.

Eight subjects participated in this study (5 female and 3 male with a mean age of 26±4 years). Each subject's near point of convergence (NPC) was measured by having an examiner slowly bring the tip of a pen towards the subject along his midline [36]. When the subject could no longer maintain fusion, the distance from the subject's orbit to the pen tip was recorded in cm as the NPC. The NPC was measured twice and averaged. All subjects had a normal near point of convergence (NPC) of less than 6 cm. Binocular vision was assessed by the Randot Stereopsis Test (Bernell Corp., South Bend, IN, USA). All subjects had normal binocular vision defined as better than 70 seconds of arc. Six of the subjects were emmetropes and two were corrected to normal refraction where the average prescription among these myopes was −1D. These two subjects wore their corrective refraction during the experiment. All subjects were right handed. None of the subjects had a history of brain injury or other neurological disorders. Subjects participated in an eye movement experiment prior to functional scanning. Each subject's eye movements were recorded to ensure the subject understood the task. All subjects were able to perform the task requested.

### Materials and Apparatus

Images were acquired using a 3.0 Tesla Siemens Allegra MRI scanner with a standard head coil (Erlangen, Germany). Visual stimuli were a set of non-ferrous light emitting diode (LED) targets that formed a line 5 cm in height by 2 mm in width located at three positions.

Eye movements were recorded using an infrared (λ = 950 nm) limbus tracking system manufactured by Skalar Iris (model 6500, Delft, Netherlands). All of the eye movements were within the linear range of the system (±25°). The left-eye and right-eye responses were calibrated, recorded and saved separately for offline analysis. A custom MATLAB™ (Waltham, MA, USA) program was used for offline eye movement data analysis. Blinks were identified by the saturation of signal. Blinks were manually omitted from the eye movement traces.

### Imaging Instrumentation and Procedure

The subject was positioned supine on the gantry of the scanner with his head along the midline of the coil. All participants were instructed to limit head motion. Foam padding was used to restrict additional movement and motion correction software described below was utilized to ensure head motion did not influence the results. Ear plugs were used to reduce scanner noise by up to 30 dB while still allowing the participant to hear instructions from the operators to ensure communication during the scan. In all experiments, the radio frequency power deposition and field-switching rate were kept below levels specified by the U.S. Food and Drug Administration (FDA).

The subcortical regions were of interest in this study. Hence, all subjects were positioned so that images could be attained of the whole brain. All functional scans used a T2* weighted echo planar imaging (EPI) sequence. The imaging parameters were field of view (FOV) = 220 mm, 64×64 matrix, time of repetition (TR) = 2000 ms, time of echo (TE) = 27 ms and flip angle = 90°. The whole brain was imaged in an axial configuration where 32 slices were collected and each slice was 5 mm thick. The resolution was 3.4×3.4×5 mm. There were 70 volumes acquired per scan lasting a total of 2 minutes and 20 seconds. Between scans, the subjects confirmed they were comfortable and could perform the task. After all functional tasks, a high resolution MPRAGE (magnetization-prepared rapid gradient-echo) data set was collected. The MPRAGE imaging parameters were: 80 slices, FOV = 220 mm, slice thickness = 2 mm, TR = 2000 msec, TE = 4.38 msec, T1 = 900 msec, flip angle = 8° and matrix = 256×256 which resulted in a spatial resolution of 0.9×0.9×2 mm.

### Functional Experimental Design

The experiment followed a standard block design of fixation (no eye movement) for the ‘off’ phase compared to random eye movements for the ‘on’ phase using saccadic or vergence step stimuli. Each visual step stimulus was presented for a random duration of time between 0.5 to 3.0 seconds. Approximately 20 visual step stimuli were presented within each eye movement phase. The subject could not anticipate the timing of the visual stimulus. Subjects confirmed they were able to comfortably view the visual stimuli during the imaging session. For all experiments, only one target was illuminated at a time.

**Figure 1:**
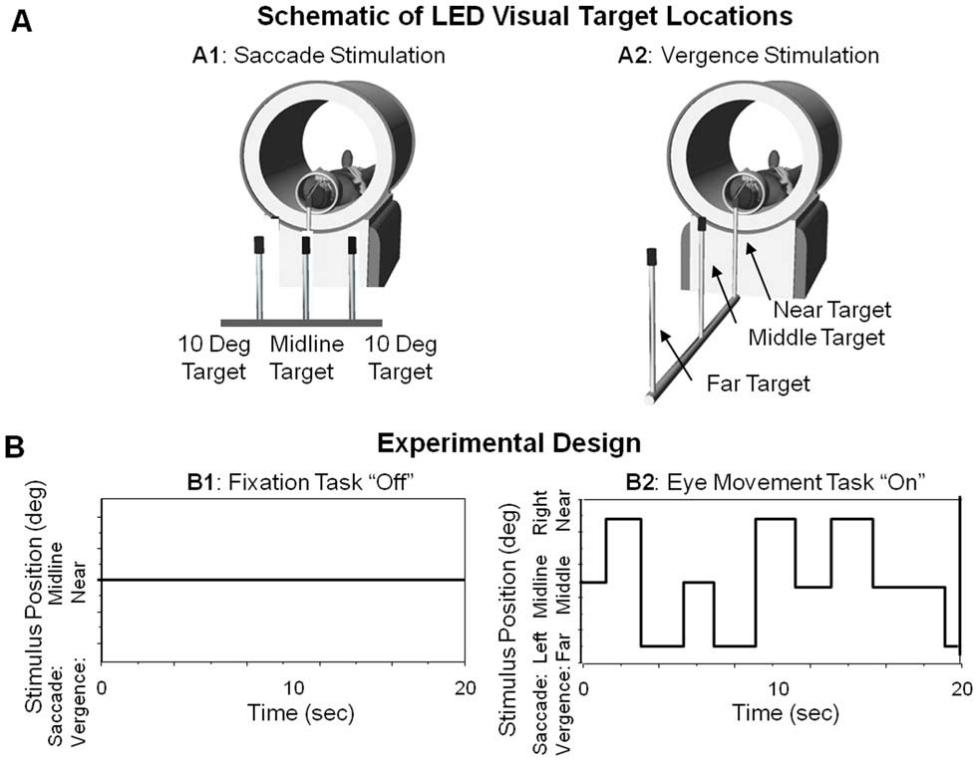
Experimental set-up and design. The schematic of the custom fMRI compatible light emitting diodes (LEDs) for the saccade (image A1) and vergence (image A2) experiments. Subjects would sustain fixation (plot B1) on either the midline target during the saccade experiment or near target during the vergence experiment for 20 seconds and then track the illuminated LEDs in a random pattern for 20 seconds (plot B2). A block design protocol is used where the “off’ stimulus is sustained fixation and the “on” stimulus is the random eye movement tracking.

The saccadic visual stimulus is shown in Figure 1A. The scanner room was darkened where the subject only saw the visual stimulus. A saccadic magnitude of 10° from midline was chosen because saccades less than 15° from midline do not evoke head motion [37]. The saccadic experiment began with fixation on the middle LED for 20 seconds, shown in Figure 1 image B1. Next, subjects would track targets that would randomly appear in three locations: 0° (midline); 10° into the left visual field; or 10° into the right visual field, Figure 1 image B2. Subjects performed tracking of saccadic step stimuli lasting for the duration of 20 seconds. This sequence was repeated for 3.5 cycles.

For vergence stimulation, subjects viewed the same LED apparatus used during the saccadic experiment, but the orientation was changed to be aligned with the subject's midline and the spacing between visual targets was adjusted to stimulate 2°, 3° and 4° of combined sustained convergence demand as shown in Figure 1 image A2. Experiments also took place in a darkened room where the subject only saw the visual stimulus. There were three vergence fixation points, 2°, 3° and 4° centered along the subject's midline to produce symmetrical vergence step stimuli. The maximum vergence step stimulus was a 2° disparity change which was chosen due to the physical constraints of the imaging center and to decrease the occurrence of saccades within the symmetrical vergence response [38], [39], [40]. For the random phase, the time when the next target was displayed was randomized between 0.5 to 3 seconds in duration.

A total of three saccade and three vergence experimental trials were collected in case head motion was a problem which was not the case within this data set.

### Data Analysis

#### Individual Subject Analysis using a Data Driven Reference Vector

Data were analyzed with AFNI (Analysis of Functional NeuroImages) software [41]. All the scans were first registered and motion corrected. A minimum least-square image registration method available in AFNI was utilized to detect and correct for the presence of any motion-induced changes on the 3D image space. Six parameters were monitored to determine whether head motion was a problem within our data set. Three parameters indicated the movement within each plane (anterior to posterior, right to left, and inferior to superior, calculated in mm) and three parameters indicated the amount of rotation about the three orthogonal axes (yaw, pitch and roll, calculated in degrees). A recent comparison of several software packages found that the AFNI image registration algorithm was both reliable and fast in comparison with other software [42]. The least-square image registration method employed in this study used the fourth image in each data set as a reference and the motion parameters were estimated for the time-series set. After motion correction, individual anatomical and functional brain maps were transformed into the standardized Talairach-Tournoux coordinate space [43].

**Figure 2:**
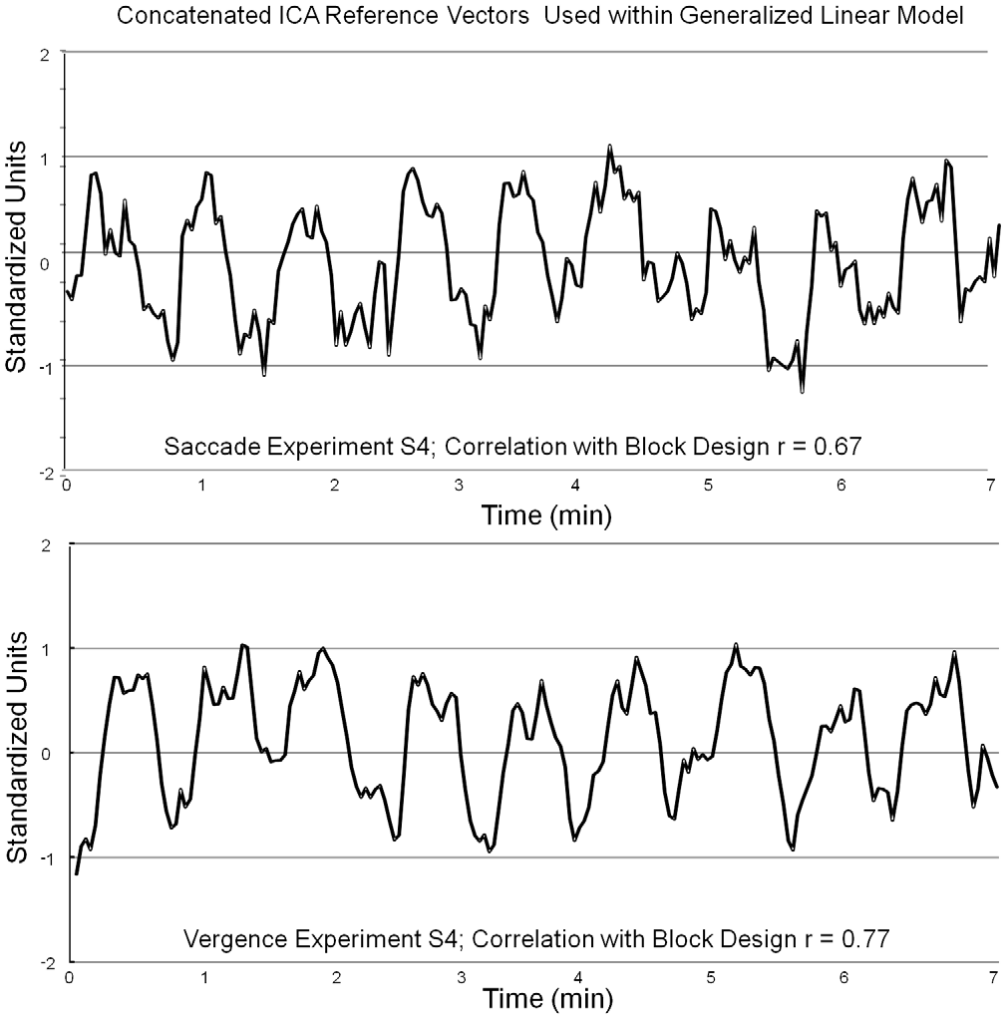
Typical reference vectors from one subject (S4) from the saccadic data set (upper plot) and the vergence data set (lower plot). The source signals have a high Pearson correlation coefficient with the experimental block design (r = 0.67 for the saccade experiment and r = 0.77 for the vergence experiment).

The hemodynamic response can vary due to age [44], [45], trauma [46], [47], fatigue [48] and / or physiological variations [49]. Due to the variations in the hemodynamic response function, a data driven independent component analysis was used to obtain a reference vector corresponding to the experimental stimulus [50], [51], [52], [53], [54], [55], [56], [57], [58], [59]. Probabilistic independent component analysis available through the MELODIC (Multivariate Exploratory Linear Optimized Decomposition into Independent Components) software from FSL was used to calculate the independent signal sources [60] for each subject. The signal source that had the greatest Pearson correlation coefficient with the experimental block design was the reference vector used to correlate each voxel within our data set during an individual subject analysis. A representative example of typical source vectors from subject S4 for the saccade and vergence experiment are shown in Figure 2.

The fMRI blood oxygenation level dependent (BOLD) signal analyzed using a general linear model (GLM) method has been reported to be correlated to direct neuronal measurements [61], [62]. Hence, the fMRI time series data within this study were analyzed with a GLM where each voxel of the entire brain was correlated with a hemodynamic model calculated using independent component analysis for each individual subject described above. Using the GLM analysis, only data that attained a minimum threshold of functional activity corresponding to a z-score of 2.0 (two tail p = 0.05) were further analyzed.

### Group Analysis

Both individual and group analyses were performed. To facilitate comparison between the vergence and saccade data sets the individual subject spatial maps were averaged. All data were first analyzed individually to observe the regions of interest (ROIs) significantly activated during the experiments. The ROIs are described below. Only ROIs that were functionally activated in all eight subjects are reported within this study.

### Regions of Interest

The functional activity for the saccadic network is well established and is reviewed in several papers [1], [7], [63]. Hence, we hypothesized that our fixation versus random saccade eye movement experiment would provoke activation within the frontal eye fields (FEF), the supplementary eye field (SEF), the dorsolateral prefrontal cortex (DLPFC), the parietal eye fields (PEF), the anterior and posterior cingulate cortex and the cerebellum during the saccadic experiment. Functional MRI studies have shown that the saccade related area of FEF is localized in the upper portion of the anterior wall of the precentral sulcus [64]. It is described in a recent review paper as being in the vicinity of the precentral sulcus and / or in the depth of the caudalmost part of the superior frontal sulcus [10]. The human SEF is located on the medial surface of the superior frontal gyrus, in the upper part of the paracentral sulcus [65]. The dorsolateral prefrontal cortex is located within Brodmann Areas (BA) 46 and 9 [66]. The parietal eye field is located in the lateral intraparietal area [63]. The anterior and posterior cingulate cortexes are located in Brodmann Areas (BA) 24 and 23 respectively [63]. These regions were initially investigated as well as other areas within the brain. The individual time series from regions shown within the results are filtered with a first order Butterworth filter using a cutoff frequency of 0.1 Hz, implemented in MATLAB™.

### Statistical Analysis

The combination of the individual voxel probability threshold and the cluster size threshold (11 voxels rounded to a volume of 650 mm^3^ for our data set) yielded the equivalent of a whole-brain corrected for multiple comparison significance level of α<0.001. The cluster size was determined using the AFNI AlphaSim program [67]. This program estimates the overall significance level by determining the probability of false detection through Monte Carlo simulation. Through individual voxel probability thresholding and minimum cluster size thresholding, the probability of false detection is determined from the frequency count of cluster sizes. The program is based on the assumption that the underlying population of voxel intensity has a normal distribution. Our simulation used 10,000 Monte Carlo iterations, assumed a cluster connection of the nearest neighbor, voxel dimension of 3.4×3.4×5 mm and sought a significance level of 0.001. Hence, a cluster size of 650 mm^3^ or greater corresponded to p<0.001 corrected for multiple comparisons. The functional data are displayed as a z-score shown in the figure scale bar. Individual maps of t-statistics were smoothed with a Gaussian kernel of 6 mm full-width and half-maximum to account for inter-individual anatomical variation [68], [69], [70].

We hypothesize that the vergence and saccade circuits will show some spatial differentiation. Specifically, we hypothesize that the vergence FEF will be adjacent and directly anterior to the functional activity of the saccadic FEF as is reported in single cell recordings in primates [11]. The null hypothesis would be that no difference in the signal amplitude would be observed between the vergence and saccade data sets. Hence, to determine whether significant spatial differences existed between the saccade and vergence data sets, the beta weights from the general linear model were compared with a paired *t*-test of the eight individual subjects in a voxel-wise basis to create a statistical significance spatial map. Data were thresholded for an absolute T-value greater than 2.3 (two-tailed p-value = 0.05). The statistical difference spatial maps are displayed using the scaled T-value as the color overlay upon standardized anatomical images to show the spatial location of significantly different areas of activation.

Using the paired *t*-test spatial maps, we observed which ROIs were significantly different between the vergence and saccade datasets. For the ROIs where significant spatial differences were observed, the results are reported using the individual subject data. All other regions of interest that were not significantly different between the vergence and saccade dataset are reported using group data.

Functional spatial maps are displayed using the Computerized Anatomical Reconstruction and Editing Tool (Caret) Kit [71].

### Representation of Time Series Signal

The time series from the functional images are displayed as the percent of signal change from the baseline. The baseline was calculated as the average of the five data points with the smallest magitude.

## Results

Six motion related parameters were computed and corrected for each subject during each of the scans. The largest average degree of rotation was 0.1°±0.1 and 0.2°±0.1 in the pitch direction for the saccade and vergence data sets respectively. The largest average amount of movement within a plane was 0.3±0.2 mm and 0.3±0.3 mm in the inferior to superior plane for the saccade and vergence data sets respectively. This motion was much less than one voxel. Hence, head motion was not problematic within these data sets. Thus, all data were utilized for this analysis.

**Figure 3:**
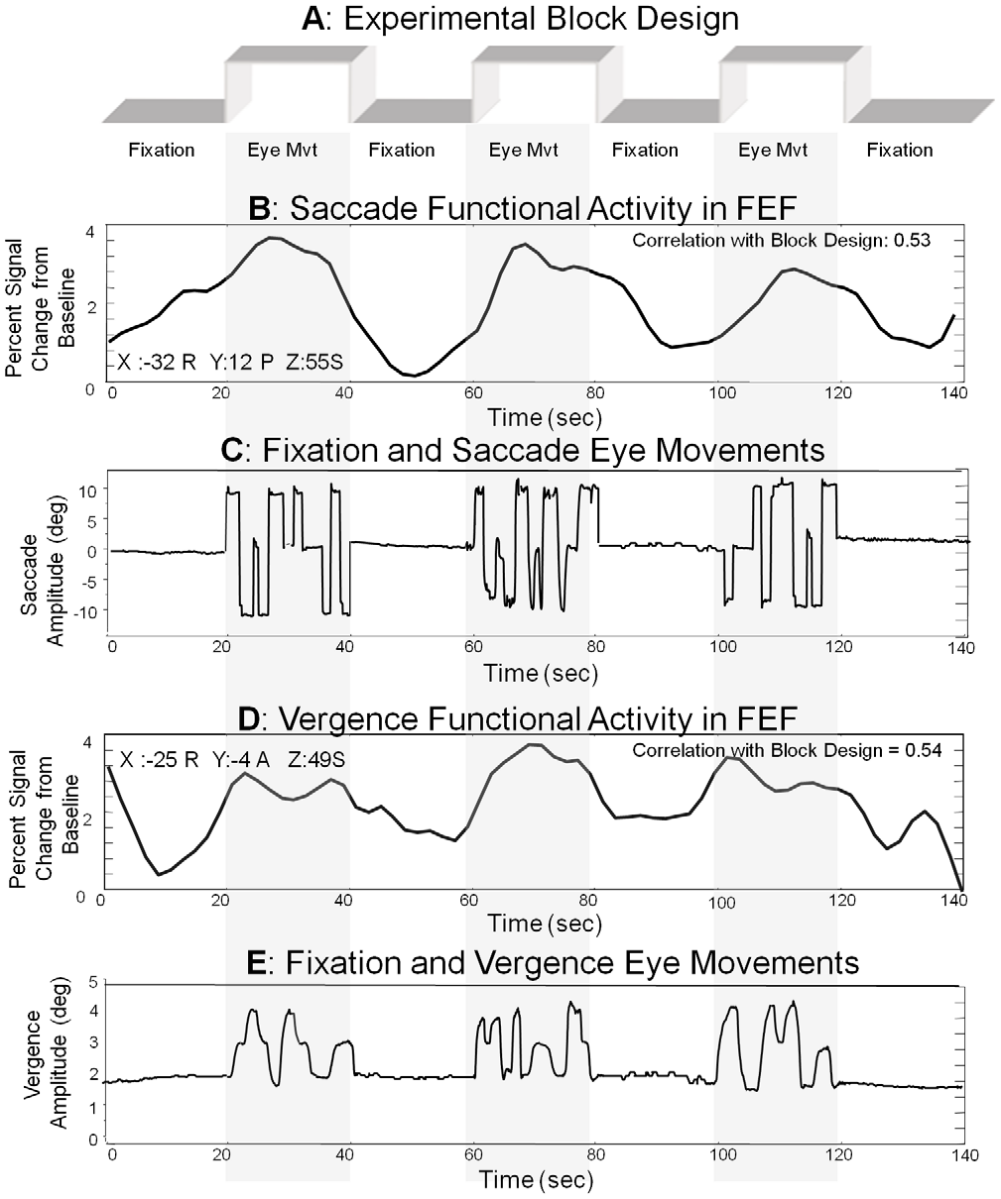
Experimental block design of 3.5 cycles alternating between fixation and eye movements (Plot A). Fixation and saccadic eye movements to targets 10 degrees into the left or right visual field or along midline plotted as position (deg) as a function of time (sec) (Plot B). Functional activity within FEF during saccadic stimulation plotted as percent signal change from baseline as a function of time (sec) (Plot C). Fixation and vergence eye movements to targets 2, 3, or 4 degrees along midline plotted as position (deg) as a function of time (sec) (Plot D). Functional activity within FEF during vergence stimulation plotted as percent signal change from baseline as a function of time (sec) (Plot E).

Typical eye movements and the corresponding functional activity from the frontal eye fields are shown in Figure 3. Data are displayed with the experimental block design. Eye Movements are plotted as position (deg) as a function of time (sec). Saccadic eye movements reach the next target sooner than the vergence eye movements. Hence, the peak velocities between these movements are different. The temporal and dynamic differences are hypothesized to be generated by differences in neural substrates because both movements are generated using the same biomechanics, the lateral and medical recti muscles.

**Figure 4:**
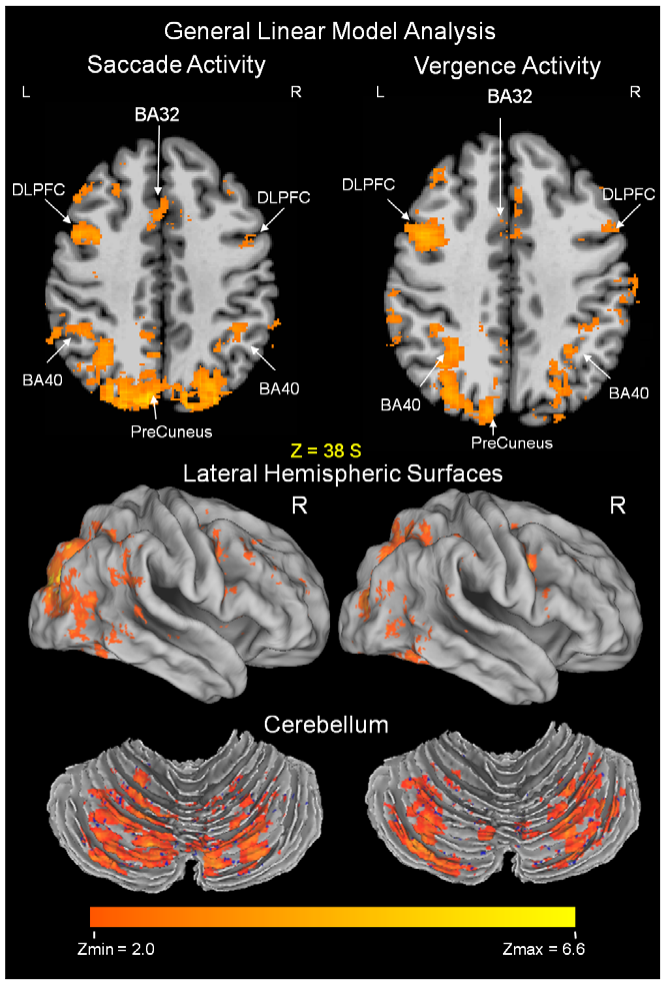
Functional activation for the group analysis of fixation versus random eye movements for the saccade (left side) and the vergence data set (right side) showing typical commonality. DLPFC = dorsolateral prefrontal cortex and BA = Brodmann Area. The number of mm above the bicommissural plane is indicated. The functional activation is denoted by the scale bar as a z-score from a minimum of 2.0 to a maximum value of 6.6. Data are overlaid onto a standardized Talairach-Tournoux normalized image. Semi-inflated images of the functional activity within the lateral hemispheric surface and cerebellum are displayed using Caret software.

**Figure 5:**
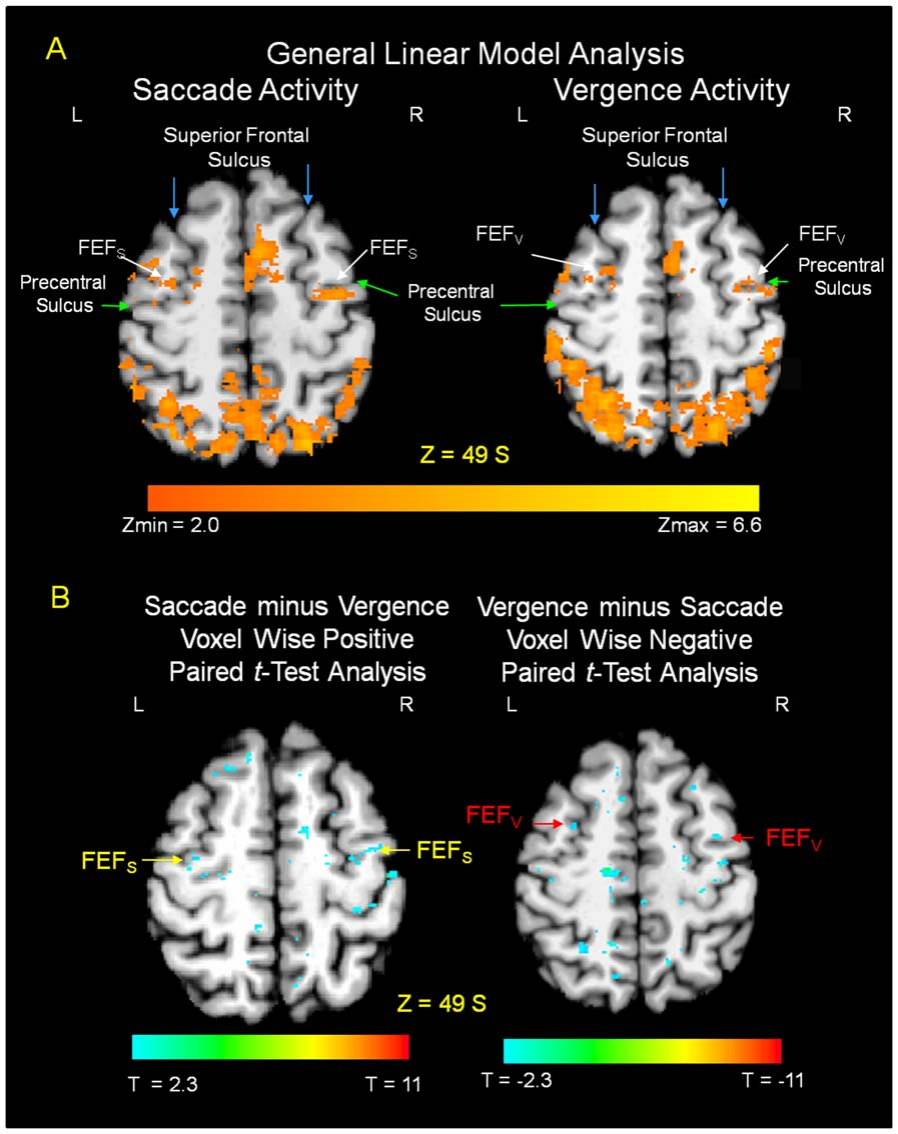
Axial images showing differentiation between the functional activity of the frontal eye fields (FEF) from saccade (left) and vergence (right) eye movements. Functional activity using the GLM analysis is shown in Figure 5A. The voxel wise positive and negative paired *t*-tests show significant differentiation between FEF for vergence and saccades, Figure 5B. The GLM analysis reports activity using the scale bar of a z-score from 2.0 to 6.6. The paired *t*-tests using the beta weights from the GLM analysis reports significant differences from T = ±2.3 to ±11 (two-tailed p-value = 0.05 to p<0.0001). Functional activity and paired *t*-test significant differences are overlaid onto Talairach-Tournoux normalized axial structural images. The axial slice is 49 mm superior to the bicommissural plane for all images. L: left; R: right. The superior frontal sulcus is denoted with blue arrows and the precentral sulcus is denoted with green arrows in Figure 5A. The significant differences within FEF are denoted with red arrows for vergence (FEFv) and yellow arrows for saccades (FEFs) in Figure 5B.

Data were first analyzed individually to determine how many of the eight subjects showed activation for a given anatomical location. Only areas that showed significant activation for all subjects are included in the results. The averaged group functional activity from the eight subjects performing the fixation versus a random saccadic oculomotor task is shown in Figures 4 and 5, left portion of the figures. Figures 4 and 5 show an axial slice displaying the anatomy of functional activity. Figure 4 also shows semi-inflated views of the lateral hemispheric surfaces and the cerebellum. Table 1 lists the peak activation with Talairach-Tournoux coordinates for a given anatomical location of the averaged subject data set with the corresponding z-score and Brodmann Area (BA) from the saccadic task. For the saccadic functional activation induced from the fixation versus random eye tracking oculomotor visual tasks, activity is observed in the vicinity of the superior frontal sulcus (denoted with a blue arrow Figure 5) and precentral sulcus (denoted with a green arrow Figure 5), also defined as the frontal eye fields [10]. Functional activity is also observed in the medial frontal gyrus, referred to as the supplementary eye field; the dorsolateral prefrontal cortex; the ventral lateral prefrontal cortex; the intraparietal sulcus, referred to as the parietal eye field or Brodmann Area 40 [63]; the cuneus; the precuneus; the anterior and posterior cingulates; and the cerebellar vermis. Similar areas were activated within the vergence data as shown in Figures 4 and 5 as well as Table 2.

**Table 1:** Average peak activation of the fixation versus random saccadic oculomotor task in Talairach-Tournoux coordinates with the level of significance denoted as a z-score.

Region	Brodmann Area	X (mm)	Y (mm)	Z (mm)	z-score
Frontal Eye Field, Superior Middle Frontal Gyrus, Precentral Gyrus	8/6	−36L	−9P	50S	3.4
		30R	−5P	48S	2.4
Supplementary Eye Field, Medial Frontal Gyrus	6	1R	−2P	53S	2.7
Dorsolateral Prefrontal Cortex	9	−33L	2A	36S	3.4
		36R	4A	34S	2.5
Anterior Cingulate/ Cingulate Gyrus	24/32	−2L	14A	38S	2.8
Inferior Ventral Lateral Prefrontal Cortex, Inferior Frontal Gyrus, precentral Gyrus	45/47	−26L	17A	1S	3.7
		41R	11A	2S	2.7
Parietal Eye FieldInferior Parietal Area	40	−33L	−53P	48S	3.6
		29R	−54P	41S	2.8
Cuneus, Lingual Gyrus	17/18	8R	−70P	12S	6.4
Precuneus	7	−5L	−73P	42S	4.4
Superior Parietal Area	7	−30L	−65P	50S	4.3
		26R	−70P	50S	4.7
Posterior Cingulate	31	1R	−67P	23S	2.6
	30	−7L	−65P	8S	5.0
		6R	−59P	8S	3.9
	29	0	−40P	18S	3.2
Cerebellar Vermis IV/V		6R	−61P	−5I	4.1

For the x axis: positive is right (R) and negative is left (L); for the y axis: negative is posterior (P) and positive is anterior (A); and for the z axis: positive is superior (S) and negative is inferior (I).

**Table 2:** Average peak activation of the fixation versus random vergence oculomotor task in Talairach-Tournoux coordinates with the level of significance denoted as a z-score.

Region	Brodmann Area	X(mm)	Y(mm)	Z(mm)	z-score
Frontal Eye Field, Superior Middle Frontal Gyrus, Precentral Gyrus	8/6	−32L	6A	49S	2.3
	8/6	24R	2A	50S	2.5
Supplementary Eye Field, Medial Frontal Gyrus	6	−3L	7A	49S	2.9
Dorsolateral Prefrontal Cortex	9	−48L	10A	35S	2.6
		43R	4A	35S	4.7
Anterior Cingulate/ Cingulate Gyrus	24/32	2R	20A	38S	2.2
Inferior Ventral Lateral Prefrontal Cortex, Inferior Frontal Gyrus, precentral Gyrus	45/47	−50L	13A	5S	2.3
		53R	17A	7S	2.9
Parietal Eye Field					
Inferior Parietal Area	40	−26L	−54P	44S	3.5
		29R	−52P	46S	4.2
Cuneus, Lingual Gyrus	17/18	12R	−92P	−3I	4.8
Precuneus	7	11R	−79P	37S	3.5
Superior Parietal Area	7	−32L	−55P	51S	3.5
		26R	−55P	52S	4.4
Posterior Cingulate	31	1R	−66P	22S	2.5
	30	−7L	−65P	9S	3.1
		5R	−65P	7S	3.8
	29	−12L	−53P	−1I	2.1
		7R	−41P	5S	2.5
Cerebellar Vermis IV/V		−9L	−43P	2S	2.2
Midbrain		8R	−20P	−4I	2.4

For the x axis: positive is right (R) and negative is left (L); for the y axis: negative is posterior (P) and positive is anterior (A); and for the z axis: positive is superior (S) and negative is inferior (I).

**Figure 6:**
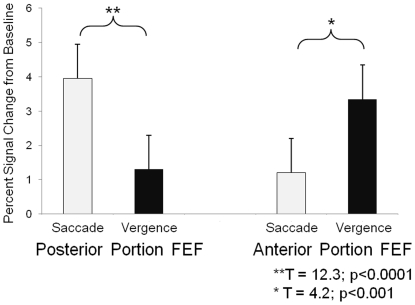
Percent signal change from baseline within the posterior (left) and anterior (right) portions of the frontal eye fields (FEF). Significantly more signal change is observed within the posterior portion of FEF in the saccade compared to the vergence data set. Significantly more signal changes is observed within the anterior portion of FEF in the vergence compared to the saccade data set.

Although the functional activation from saccadic and vergence visual stimuli had many shared neural resources, we did observe differentiation within the frontal eye fields, Figures 5 and 6 and Table 1 compared to Table 2. Specifically, group peak activation for the fixation versus vergence eye movement task was anterior, quantified as Talairach-Tournoux coordinates 32L, 6A, 49S and 24R, 2A, 50S (Table 2), to average peak bilateral activation of the saccadic task quantified as Talairach-Tournoux coordinates 36L, 9P, 50S and 30R, 5P, 48S (Table 1). The individual subject peak activation is summarized in Table 3 where we also observed on an individual basis that the peak activations in the frontal eye fields were anterior to the activation within the saccade data. We then conducted a voxel-wise paired *t*-test using the beta weights from the general linear model to determine whether the cortical activation was significantly more anterior. Results are shown in Figure 5.

**Table 3:** Individual subject analysis of saccade and vergence FEF.

Subject	Saccadic Task				Vergence Task			
	X(mm)	Y(mm)	Z(mm)	z-score	X(mm)	Y(mm)	Z(mm)	z-score
1	−38L	−10P	49S	6.4	−30L	12A	57S	2.8
	40R	1A	48S	6.6	23R	5A	58S	3.9
2	−32L	−7P	49S	9.5	−25L	13A	51S	3.8
	36R	−6P	50S	5.7	33R	17A	49S	4.2
3	−33L	−12P	52S	6.7	−23L	12A	46S	4.0
	29R	−5P	52S	5.7	25R	16A	47S	6.6
4	−31L	−5P	53S	4.3	−27L	11A	47S	4.5
	25R	−6P	58S	3.8	37R	5A	41S	11.7
5	−31L	−10P	54S	7.4	−23L	10A	50S	3.7
	36R	−16P	54S	5.0	26R	8A	57S	5.5
6	−34L	−7P	46S	2.6	−21L	13A	50S	4.3
	32R	−2P	45S	4.8	22R	7A	54S	4.4
7	−30L	1A	47S	5.1	−30L	10A	47S	2.9
	30R	1A	52S	8.3	19R	7A	51S	3.4
8	−29L	−6P	53S	4.2	−21L	11A	40S	4.0
	34R	−7P	53S	7.4	27R	10A	38S	5.1
Average ± Standard Deviation	−32L±3	−7P±4	50S±3	5.8±2.2	−25L±4	12A±1	49S±5	3.8±0.6
	33R±5	−5P±5	52S±4	5.9±1.5	27R±6	9A±5	49S±7	5.6±2.7

Average peak activation of the fixation versus random oculomotor task in Talairach-Tournoux coordinates with the level of significance denoted as a z-score. For the x axis: positive is right (R) and negative is left (L); for the y axis: negative is posterior (P) and positive is anterior (A); and for the z axis: positive is superior (S) and negative is inferior (I).

Figure 5A shows the functional activity from the fixation versus the random saccadic eye movement task as well as the fixation versus the random vergence eye movement task where the FEF (white arrows), the precentral sulcus (green arrows) and the superior frontal sulcus (blue arrows) are labeled. The functional activity for vergence is anterior to a similar saccadic task. Figure 5B shows the statistical spatial maps of the saccade minus vergence paired *t*-test data sets (defined as the positive values) and statistical spatial maps of the vergence minus saccade paired *t*-test data sets (defined as the negative values). Significant spatial differentiation was observed. We then sought to evaluate the correlation of the underlying time series with the experimental block design.

One subject's time series signals from the frontal eye field for one trial (2 min 20 sec) are assessed in Figure 6. The time series with the maximum correlation with the block design (a square wave of ‘off’ and ‘on’ stimuli for 3.5 cycles) has a Pearson correlation coefficient of r = 0.64. This signal is from Talairach-Tournoux location 33R, 11P, 56S within the frontal eye field (FEF) from the saccade data set. The time series with the maximum correlation with the block design from the frontal eye field within the vergence data set has a correlation of r = 0.54 (25R, 4A, 49S, in Talairach-Tournoux coordinates). Hence, the time series are highly correlated with the experimental task. Figure 6 quantifies the time series from the anterior and posterior portions of the FEF. The percent signal change from baseline was computed from the time series of matched Talairach-Tournoux coordinates. Data were compared using a paired *t*-test. Within the posterior portion of FEF, the percent signal change from baseline was significantly greater in the saccade compared to the vergence data set (T = 12.3; p<0.0001). Within the anterior portion of FEF, the percent signal change from baseline was significantly greater in the vergence compared to the saccade data set (T = 4.2; p<0.001).

**Figure 7:**
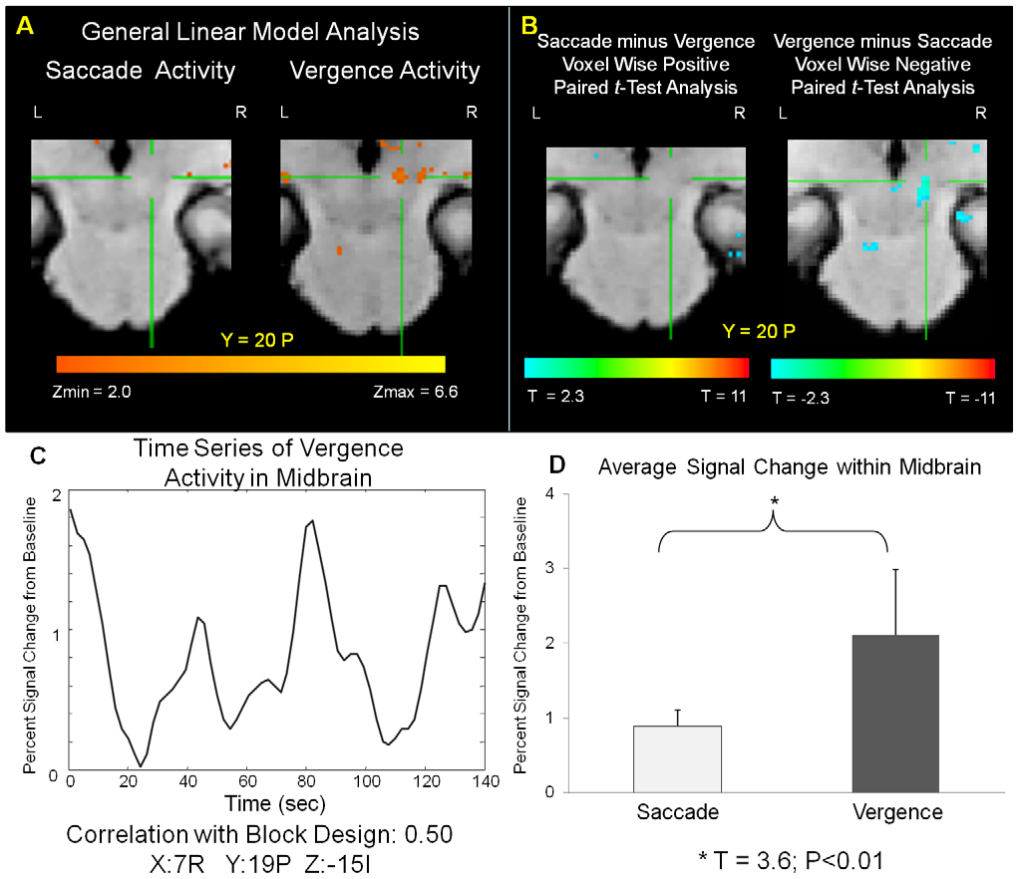
Functional activity within the midbrain using a GLM analysis (Plot A). Positive and negative paired *t*-test statistical spatial map showing differentiation between the midbrain for the fixation versus random saccadic and vergence tasks identified via the cross hair. A positive T value is for the saccade minus vergence data set and a negative T value is for the vergence minus saccade data set (Plot B). Typical time series signal from the midbrain which has a correlation of 0.5 with the block design (square wave) (Plot C). Talairach Tournoux coordinates are: 7 R, 19 P and 15 I. Comparison of the percent signal change from baseline of the same time series signals from the saccade and vergence data sets within the midbrain (Plot D).

Differentiation was also observed subcortically within the midbrain, Figure 7A. We observed activity within the vergence data set (Figure 7A right) but not within the saccade data set (Figure 7A left). An individual subject analysis was conducted and the Talairach-Tournoux coordinates with the z-score of peak activation is summarized in Table 4. A paired *t*-test analysis using spatial maps was performed on the subcortical regions to determine whether these activations were significantly different between the saccade and vergence data sets, Figure 7B. The area within the cross hair shows that this region of interest is significantly different between the vergence and the saccadic dataset. A typical filtered time series signal of the midbrain from the vergence data set is shown in Figure 7C. This signal has a correlation of r = 0.50 with the block design (square wave) and is located at Talairach-Tournoux location 7R, 13P, 15I. The midbrain functional activity within the vergence data set is highly correlated with our experimental task. Furthermore, when comparing the same subcortical locations within the saccade and vergence data sets, significant differences between the percent signal change from baseline are observed assessed using a paired *t*-test. Specifically, there is a greater percent signal change from baseline in the vergence data set compared to the saccade data set, (T = 3.6, p<0.01). Results are shown in Figure 7D.

**Table 4:** Individual subject analysis of the midbrain from the vergence data set.

Subject	X(mm)	Y(mm)	Z(mm)	z-score
1	6R	−24P	−10I	4.8
2	10R	−19P	−12I	2.6
3	12R	−24P	−6I	4.7
4	5R	−27P	3S	4.0
5	5R	−13P	−8I	4.2
6	8R	−22P	−5I	2.9
7	6R	−19P	−7I	3.5
8	6R	−10P	3S	3.8
Average ± Std	7R±3	−20P±6	−5I±6	3.8±0.8

Table 5 highlights some specific, significant differences observed within the paired *t*-tests spatial maps shown in Figure 5 (identified via yellow arrows for the saccade data and red arrows for the vergence data) and Figure 7 (identified via the cross hairs). Table 5 summarizes the z-score of a specific Talairach-Tournoux location of activation within the frontal eye fields for saccades and vergence and the midbrain for the vergence data set. These specific Talairach-Tournoux locations highlight significant differences between the saccade and vergence data sets quantified via a two tailed positive or negative T value greater than 2.3.

**Table 5:** Saccade minus vergence data sets / positive paired *t*-test and vergence minus saccade data sets / negative paired *t*-test statistics showing differentiation between FEF and midbrain in comparing fixation versus random saccade and vergence tasks.

Region	Talairach -Tournoux Stereotactic Coordinates			z-score in Saccade Data set	z-score in Vergence Data set	Paired *t*- test T value
	X (mm)	Y (mm)	Z (mm)			
**FEF**(Activity in Saccade data set)	−36L	−9P	50S	3.4	<1	2.7
	30R	−5P	48S	2.4	<1	2.4
**FEF**(Activity in Vergence data set)	−32L	6A	49S	<1	2.3	−2.3
	24R	2A	50S	<1	2.5	−2.3
**Midbrain**(Activity in Vergence Data set)	8R	−20P	−5I	<1	2.6	−4.3

Peak activation per subject of the fixation versus random vergence oculomotor task in Talairach-Tournoux coordinates with the level of significance denoted as a z-score. For the x axis: positive is right (R) and negative is left (L); for the y axis: negative is posterior (P) and positive is anterior (A); and for the z axis: positive is superior (S) and negative is inferior (I).

## Discussion

### Significant Spatial Differences between Saccade and Vergence Data Sets

The activation within the frontal eye fields (FEF) showed significant spatial differences between the saccade and vergence data sets where the vergence activation was located directly anterior to the saccadic activation. The location of the FEF is located in the vicinity of the intersection of the precentral sulcus and the superior frontal sulcus [10], [72], [73], [74], [75]. One human functional imaging study of vergence eye movements used positron emission tomography (PET) but did not observe any significant signals within the frontal lobe, which they attribute to a limitation of the PET instrumentation [76].

There are four non-human primate single cell electrophysiology studies that investigated the influence of disparity in FEF using symmetrical step stimuli [11], near and far targets [77], and smooth sinusoidal tracking stimuli [78], [79]. The first study of symmetrical steps is the most relevant to our present investigation because the visual stimuli are the same. When studying symmetrical vergence step stimuli in non-human primates, Gamlin and Yoon (2000) report differentiation within the FEF. Cells that encode for symmetrical vergence stimuli were located adjacent and anterior to cells that encoded for saccadic stimuli [11]. Most of these vergence cells (28 out of 34) did not significantly change their activity during conjugate (saccade or smooth pursuit) eye movements. They also tested monocular vision to determine whether the cells modulated their activity correlated with a motor signal rather than a sensory retinal disparity input. Their data support that these cells are “more closely related to the movement than to the retinal disparity of the target that elicited it” [11]. Our study of functional activity in humans using fMRI support similar findings that the vergence activity to symmetrical vergence steps within the FEF was located adjacent and significantly anterior to functional activity evoked using fixation versus random saccadic eye movements.

The other non-human electrophysiology studies of FEF are not as directly related to our present study. One reports that approximately two-thirds of the cells that traditionally modulate their activity during saccadic movements are broadly tuned for near or far disparity signals and hence these cells do carry information about depth [77]. The last two studies investigated smoothly moving targets in depth (smooth vergence movement) and within the frontal plane (smooth pursuit movement). Reviewing the two studies, the results showed that 63% to 66% of the neurons studied within the caudal portion of FEF encoded for both smooth pursuit and smooth vergence tracking signals, 21% to 25% encoded only smooth pursuit signals and 17% to 9% responded only during smooth vergence tracking [78], [79]. These three studies on FEF all tested different stimuli compared to the symmetrical step studied within our present investigation.

The midbrain within the brain stem has also been identified as a region that encodes specifically for vergence and projects directly to the oculomotor neurons [13], [14]. Three types of cells have been identified: vergence tonic cells (also called positioning encoding cells), vergence burst cells (also called velocity encoding cells), and burst-tonic cells. One study states “conjugate and vergence signals are generated independently and are combined at the extraocular motoneurons” [80]. Our data support differentiation within the midbrain where functional activity was observed in the vergence data set but not in a similar location for the saccade data set. Some cellular differentiation between the saccade and vergence systems is expected. Patients with internuclear ophthalmoplegia have a loss of saccadic or adduction movements but vergence or abduction movements are preserved [81], [82].

### Shared Neural Resources between Saccade and Vergence Data Sets

Several behavioral studies discuss the nonlinear interaction between the saccadic and vergence systems [18], [19], [20], [21], [22]. Our laboratory and other investigators have published that even when symmetrical vergence stimuli are presented to a subject, many of the responses contain saccades [38], [39], [40]. Similarly, studies have shown that with saccadic movement, a transient divergent and then convergent movement is observed [83], [84]. Therefore, it would be expected that the vergence and saccade oculomotor systems would share some neural resources. Our results support many shared neural areas in terms of similar amplitudes and spatial extent of functional activity.

Specifically, we saw similar activity within the supplementary eye fields when comparing the vergence and saccade data sets. For saccades, the SEF has been identified as an area involved in gain control [85], attention [86], and in the production of an error signal [73]. We saw activation in SEF for both the fixation versus random eye movement tasks utilizing vergence or saccade responses which may be from high-level processes, as supported in other studies that may potentially influence both systems. The dorsolateral prefrontal cortex has been described as a brain region that supports attention, planning, spatial orientation and behavioral restraints [9], [87], [88]. Activation within the dorsolateral prefrontal cortex was also similar between the vergence and saccade data sets and may in part be activated from these higher-level cognitive functions. The ventral lateral prefrontal cortex also showed similar areas of activation within this study. It has been implicated in working memory and task switching [89], [90]. Both these cognitive functions were evoked to follow the experimental protocol within this study.

Both the anterior and posterior cingulates stimulated similar functional activity for fixation versus random eye movements utilizing saccade and vergence stimuli. Several studies of saccadic eye movements support that the anterior cingulate is involved in regulating error [91], [92], [93], [94] where the execution of saccades within our study would also need to regulate an error signal. Similar studies for vergence are not available. The posterior cingulate cortex (PCC) has been suggested to be involved in visuospatial encoding and attention while studying saccadic movements from primates [95], [96], [97]. The saccadic and vergence activation within the PCC of this study may also in part be due to visuospatial encoding and attention.

Several studies support that the parietal lobe is involved in visual attention when studying saccadic eye movements [25], [26], [27]. Two recent papers studying hand reaching in depth support the hypothesis that a disparity signal is encoded within the parietal area [28], [29]. Functional imaging vision studies of humans support a disparity signal is present in the parietal lobe [76], [98], [99], [100] as do single cell recordings from primates [101], [102]. The present study supports that the parietal lobe is functionally active during both saccadic and vergence eye movements.

The cerebellum also showed similar activation behaviors between the saccadic and vergence data sets. Many studies from just the last two years support the concept that the cerebellum is involved in saccadic eye movements and is responsible for processing error that is used for motor learning [30], [31], [32]. The cerebellar vermis also participates in vergence eye movements as reported by dysfunctional vergence eye movements in patients with lesions [103] and primate studies [33], [34], [35]. Our data support the hypothesis that the cerebellum is involved in voluntary saccadic and vergence eye movements.

### Conclusion

Our study used a block design of fixation compared to a random eye movement task composed of steps to study the vergence system in comparison with the saccade system. Results show several shared neural resources between the vergence and saccade systems, specifically within the supplementary eye field, dorsolateral prefrontal cortex, ventral lateral prefrontal cortex, intraparietal area, cuneus, precuneus, the anterior and posterior cingulates, and cerebellar vermis. Our results support that significant spatial differentiation exists within the frontal eye fields. The functional activity unique to vergence was adjacent and anterior to the saccadic functional activity. Midbrain activation was observed within the vergence data set but not in the saccadic data set.
